# Rehabilitation Nursing Intervention Can Improve Dysphagia and Quality of Life of Patients Undergoing Radiotherapy for Esophageal Cancer

**DOI:** 10.1155/2021/3711699

**Published:** 2021-08-05

**Authors:** Xiange Zeng, Ling Li, Wenjing Wang, Lihui Zhu

**Affiliations:** ^1^Department of Gastroenterology, The Second Affiliated Hospital, Hengyang Medical School, University of South China, Hengyang, China; ^2^Digestive Endoscopy Center, The Second Affiliated Hospital, Hengyang Medical School, University of South China, Hengyang, China

## Abstract

**Objective:**

To seek the improvement of rehabilitation nursing intervention on dysphagia and quality of life of patients with esophageal cancer undergoing radiotherapy.

**Methods:**

A total of 109 patients with esophageal cancer undergoing radiotherapy were selected as research objects. According to the random number table, they were separated into the control group (CG) and intervention group (IG), with 45 cases in CG and 64 cases in IG. In CG, patients were given routine nursing intervention, while those in IG were given rehabilitation nursing intervention. After intervention, the degree of acute radiation injury and the improvement of swallowing function were observed to compare the self-nursing ability, quality of life, and incidence of complications between the two groups.

**Results:**

The degree of injury in CG was heavier than that in IG. The improvement of swallowing function in IG was better than that in CG. The scores of self-nursing ability and life quality in IG were higher than those in CG, with statistically significant differences (*p* < 0.05). The incidence of complications in IG was obviously lower than that in CG (*p* < 0.05).

**Conclusion:**

Rehabilitation nursing intervention can ameliorate dysphagia, improve the quality of life, and reduce the incidence of complications for patients with esophageal cancer undergoing radiotherapy. It is worthy of clinical application.

## 1. Introduction

Esophageal cancer, also known as esophagus cancer, is a malignant tumor disease that develops in esophageal epithelial tissue, mostly occurring in people over 40 years old [1]. Its early symptoms are not obvious, but patients will show progressive dysphagia and other symptoms in the middle and late stages, which seriously threatens their life health [2]. The occurrence and distribution of esophageal cancer are related to many factors, including age, gender, race, living environment, and geographical distribution [3–5]. China is a country with a high incidence of esophageal cancer. Due to the improvement of diet and living habits, the incidence of esophageal cancer in China has declined in recent years, but its mortality rate remains high [6, 7]. Because there are no obvious clinical symptoms in the early stage of esophageal cancer, 80% of sufferers have entered the middle and late stages when they are diagnosed, often missing the best opportunity to radically cure it [8].

Surgery [9], radiopharmaceutical therapy (radiotherapy) [10], and chemotherapy [11] are the basic methods to treat esophageal cancer. If timely and effective treatment measures are given at the early stage of the patient's disease development, it is highly curative, so it is very necessary to give effective surgical treatment in the early and middle stage of patients' disease development [12]. However, most patients lose the chance of operation because of the location of tumor or the rapid progress of disease, so radiotherapy needs to be given to patients with advanced disease. However, the spread of cancer cells cannot be controlled when the radiotherapy measures are implemented, so the incidence of adverse reactions is higher after treatment [13, 14], which leads to greater psychological pressure of patients, directly affects their psychological state and quality of life, and has a certain negative impact on the therapeutic effect [15, 16]. Moreover, radiation injury to esophageal mucosa is one of the most common side effects of radiotherapy, which can lead to mucosal edema, congestion, ulcer, and erosion causing patients to feel esophageal burning, esophageal swelling and pain, and then suffering from dysphagia, swallowing pain, pain behind the sternum, and other symptoms when eating [17–19]. Therefore, the implementation of scientific and effective rehabilitation training and health guidance is conducive to improve the swallowing function for esophageal cancer patients with difficulty in opening mouth after radiotherapy, so as to supplement enough nutrition and water to improve the body's resistance, which is of great significance for the rehabilitation of disease and psychology. In this study, rehabilitation nursing was mainly used to intervene patients with esophageal cancer undergoing radiotherapy and observe its application effect.

## 2. Materials and Methods

### 2.1. Research Objects

A total of 109 patients with esophageal cancer were selected as the research objects, and the patients were admitted to hospital from June 2019 to June 2020. They were randomly divided into CG and IG in accordance with the random number table. There were 45 cases in CG, including 29 men and 16 women, with an mean age of 47.68 ± 3.51 years old. There were 64 cases in IG, including 37 men and 27 women, with a mean age of 48.05 ± 3.57 years old.

Inclusion criteria are as follows: all of them met the diagnostic criteria of esophageal cancer, and all patients received radiotherapy.

Exclusion criteria are as follows: comorbid with other tumors, cognitive impairment, and severe hepatic insufficiency.

There was no statistically significant difference in baseline data in both groups (*p* > 0.05), which was comparable. In both groups, patients and their families have affixed informed consent, and this research has been ratified by the ethics committee of our hospital.

### 2.2. Methods

In both groups, patients received radiotherapy. During radiotherapy, patients in CG were provided with corresponding nursing measures according to the routine nursing process of esophageal cancer. The vital signs, blood routine, and systemic reactions of patients were closely observed, and symptomatic treatment was given in time. The ward was ventilated regularly to keep the air fresh, and the ward was kept quiet. The ward was regularly disinfected, and patients with low leucocyte or infection should be placed in protective isolation. The patient was placed in a comfortable position and kept the respiratory tract smooth.

In IG, patients were treated with rehabilitation nursing intervention. Specific measures: (1) preparatory work. Medical staff explained the importance and positive role of rehabilitation exercise to patients and their families and introduced the content of rehabilitation exercise, so that patients and their families could get a basic understanding, so as to establish a good attitude towards treatment and preparation. (2) Rehabilitation exercises: rehabilitators and nursing staff assisted patients in rehabilitation exercises, including mouth opening exercises, neck massage, oral organ coordination training, and direct feeding training. (3) Oral and pharyngeal nursing. The swallowing therapeutic apparatus with frequency of 30–80 Hz, wave width of 700 ms, and current intensity of 0–25 mA was used for treatment, and it could be adjusted according to the specific condition and tolerance of patients. The patient was treated for 30 min/time, once/d, and continued for 2 weeks. (4) Psychological nursing. When patients received radiotherapy, they needed to be enlightened by nursing staff to relieve their anxiety and fear and give appropriate psychological comfort to enhance their self-confidence to overcome the disease.

### 2.3. Outcome Measures

Mucosal injury degree: it was judged according to the grading standard of acute radiation injury of tumor cooperative organization in radiotherapy, which was divided into four grades, and the severity was decreased in turn according to the grades. Grade 4: the patient had a large area of ulcer, fistula, and perforation, and the pain was intense. Grade 3: the patient suffered from severe dysphagia with complications such as weight loss and severe dehydration. The patient needed to be fed by intravenous fluids infusion or nasal feeding. Grade 2: the patient suffered from moderate dysphagia with labor pains. The patient might take a liquid diet or rely on some anesthetic to relieve the pain. Grade 1: the patients suffered from mild dysphagia and could take semiliquid food, and the epidermal anesthetics could be used to relieve discomfort. Grade 0: there was no symptom.

Swallowing X-ray video (VFSS) was used to evaluate the improvement of swallowing function. Cure: the swallowing time was normal, without error aspiration, residual barium meal, and epiglottic vallecula. Effective: the swallowing time was shortened by more than 1/2 compared with before treatment, and there was no error aspiration, residual barium meal, and epiglottic vallecula. Ineffective: the swallowing time was shortened by less than 1/2 compared with before treatment, and there was error aspiration, residual barium meal, and epiglottic vallecula, or even the patient was unable to swallow.

Self-nursing ability: the self-nursing ability scale ESCA was applied to assess the self-nursing ability of the patients in both groups. The scale included four parts: self-concept, self-care responsibility, self-care skills, and health knowledge cognition, with a total of 43 items. Each item was scored by 1–4 grades. The higher the score, the higher the self-nursing ability.

The quality of life core scale (EORTC QLQ-C30) was applied to evaluate patients' life quality, which was divided into somatic function, role function, cognitive impairment, emotional function, and society function, with 30 items in total. The higher the score, the better the life quality of patients.

Incidence of complications: the complications mainly included esophagitis, tracheitis, esophageal fistula, and hemorrhage.

### 2.4. Statistical Methods

SPSS22.0 statistical software was applied to analyze the research data. The quantitative data were represented by mean number ± standard deviation (*x* ± *s*), and the *t*-test was applied. The enumeration data were represented by rate (%), and the *χ*^2^ test was applied. The difference was statistically significant with *p* < 0.05.

## 3. Results

### 3.1. Degree of Mucosal Injury in the Two Groups

The number of patients with grade 0 and grade 1 mucosal injury in IG was higher than that in CG, and the number of grade 2, grade 3, and grade 4 was lower than that in CG. The difference was statistically significant (*p* < 0.05), indicating that the degree of injury in CG was heavier than that in IG (Table 1).

### 3.2. Improvement of Swallowing Function in the Two Groups

Observing the improvement of swallowing function in both groups, the findings showed that the total response rate in IG (82.8%) was higher than 57.8% in CG, and the difference was statistically significant (*p* < 0.05) (Table 2).

### 3.3. Self-Care Ability of Patients in Both Groups

The self-nursing ability of patients was observed in the two groups. Before intervention, there was no obvious difference in scores of self-concept, self-care responsibility, self-care skills, and health knowledge cognition in both groups (*p* < 0.05). After intervention, the scores of patients in both groups increased, and the scores of patients in IG were obviously higher than those in CG (*p* < 0.05) (Table 3).

### 3.4. Life Quality of Patients in the Two Groups

**Figure 1:**
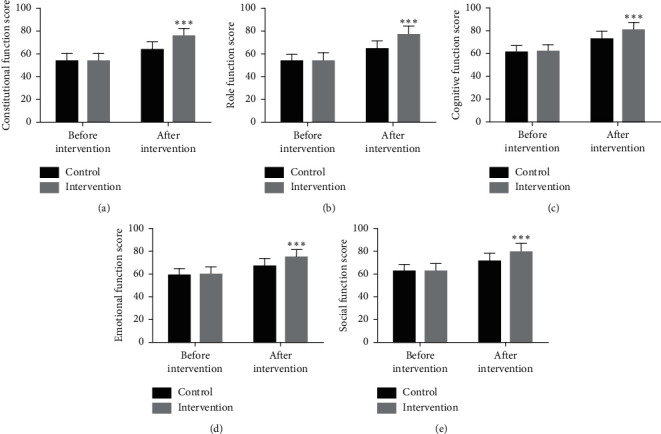
Quality of life of patients in the two groups. (a) Physical function scores of patients in the two groups. (b) Role function scores of patients in the two groups. (c) Cognitive function scores of patients in the two groups. (d) Emotional function scores of patients in the two groups. (e) Social function scores of patients in the two groups. Compared with the CG, ^*∗∗∗*^*p* < 0.0001).

There was no significant difference in EORTC QLQ-C30 scores in both groups before intervention. After intervention, the EORTC QLQ-C30 scores of the two groups were higher than those before intervention, and the scores in IG were obviously higher than those in CG (*p* < 0.05) (Figure 1).

### 3.5. Incidence of Complications in Both Groups

In IG, there were 1 case with esophagitis, 2 cases with tracheitis, 1 case with esophageal fistula, and 0 cases with bleeding, with a total of 4 cases (6.3%). In CG, there were 3 cases with esophagitis, 3 cases with tracheitis, 2 cases with esophageal fistula, and 2 cases with bleeding, with a total of 10 cases (22.2%). There was a statistical difference in the incidence of complications in both groups (*p* < 0.05) (Table 4).

## 4. Discussion

With the change of people's diet structure, the prevalence of esophageal cancer is increasing year by year. Radiotherapy has become the main treatment for esophageal cancer due to less trauma than surgery and less restrictions by surrounding tissues and trachea [20–22]. However, this treatment can lead to serious side effects and increase the psychological burden of patients [23, 24]. Therefore, it is of positive significance to carry out necessary nursing intervention for patients with esophageal cancer during chemotherapy to improve the clinical prognosis.

In this study, rehabilitation nursing was used to intervene patients undergoing radiotherapy for esophageal cancer. The results showed that the total effective rate of improving swallowing function in IG was 82.8% (53/64), significantly higher than 57.8% in the control group, which was consistent with the findings in stroke patients with hemiplegia [25]. Radiotherapy can cause different degrees of radiotherapy reaction during the treatment of esophageal cancer. The main toxic side effect is acute radiation esophagitis. The clinical manifestation of acute radiation injury is pain aggravated by eating or burning sensation behind sternum, and its incidence increases with the increase of dose, which has adverse effects on the treatment effect, life quality, and survival rate of patients. The results showed that rehabilitation nursing intervention could effectively reduce the degree of acute radiation injury. The scores of self-nursing ability and life quality in IG were higher than those in CG, and the incidence of complications in IG was 6.3% (4/64), obviously lower than 22.2% (10/45) in CG. Rehabilitation nursing is designed to make every link in the nursing process continuous and systematic and provide high-efficiency and high-quality nursing services for patients, so as to effectively relieve the psychological and physiological pressure of patients and improve the quality of nursing work. Through rehabilitation exercise, oropharyngeal care, psychological intervention, and other measures, the correct and effective rehabilitation nursing in the radiotherapy process can improve the clinical symptoms and reduce the psychological burden of patients, so that patients take the initiative to cooperate with radiotherapy to improve compliance. The results suggested that rehabilitation nursing intervention could obviously ameliorate patients' self-nursing ability and their life quality. Patients with esophageal cancer should be evaluated and take functional exercise as early as possible. Rehabilitation nursing intervention measures (the main content is to understand their condition and psychological characteristics, formulate nursing plans, give health education, rehabilitation training, specialized nursing, and psychological counseling) can significantly ameliorate the degree of swallowing difficulties of patients and prevent the development and progression of complications. This study also has some limitations. For example, the effect of patient disease types on the results of the study was not considered, and the small number of included cases may lead to bias in the results.

## 5. Conclusion

In conclusion, rehabilitation nursing intervention has a remarkable effect on dysphagia in patients undergoing radiotherapy for esophageal cancer, which can ameliorate their pain symptoms, improve the quality of life, and reduce the incidence of complications. It is worthy of popularization and application in radiotherapy.

## Figures and Tables

**Table 1:** Degree of mucosal injury in the two groups.

	Grade 0	Grade 1	Grade 2	Grade 3	Grade 4
CG (*n* = 45)	1 (2.2)	10 (22.2)	22 (48.9)	8 (17.8)	4 (8.9)
IG (*n* = 64)	9 (14.1)^*∗*^	37 (57.8)^*∗*^	15 (23.4)^*∗*^	3 (4.7)	0 (0)^*∗*^
*χ*^2^/*t*	4.4451	13.6510	7.6331	1.6881	5.9061
*P*	0.0350	0.0002	0.0057	0.1939	0.0151

^*∗*^Comparison with the CG group (*p* < 0.05).

**Table 2:** Improvement of swallowing function in the two groups.

	Cure	Effective	Ineffective	Total effective rate
CG (*n* = 45)	5 (11.1)	21 (45.7)	19 (42.2)	26 (57.8)
IG (*n* = 64)	16 (25.0)	37 (57.8)	10 (17.2)	53 (82.8)^*∗*^
*χ*^2^/*t*				8.3011
*P*				0.0040

^*∗*^Comparison with the CG group (*p* < 0.05).

**Table 3:** Self-care ability of patients in the two groups.

	Before intervention		*χ*^2^/*t*	*P*	After intervention		*χ*^2^/*t*	*P*
	CG (*n* = 45)	IG (*n* = 64)			CG (*n* = 45)	IG (*n* = 64)		
Self-concept	24.75 ± 4.26	24.94 ± 4.03	0.2367	0.8133	29.03 ± 5.12	32.68 ± 5.39^*∗*^	3.5530	0.0005
Self-care responsibility	25.37 ± 3.97	25.56 ± 4.11	0.2410	0.8100	29.77 ± 5.69	34.82 ± 5.61^*∗*^	4.6000	<0.0001
Self-care skills	25.77 ± 4.51	25.93 ± 4.22	0.1894	0.8501	29.06 ± 5.97	36.81 ± 6.23^*∗*^	6.5046	<0.0001
Health knowledge cognition	26.08 ± 4.87	26.21 ± 4.93	0.1362	0.8919	30.19 ± 6.06	36.49 ± 6.17^*∗*^	5.2871	<0.0001

^*∗*^Comparison with the CG group after intervention (*p* < 0.05).

**Table 4:** Incidence of complications in the two groups.

	Esophagitis	Tracheitis	Esophageal fistula	Hemorrhage	Overall incidence
CG (*n* = 45)	3 (6.7)	3 (6.7)	2 (4.4)	2 (4.4)	10 (22.2)
IG (*n* = 64)	1 (1.6)	2 (3.1)	1 (1.6)	0 (0)	4 (6.3)^*∗*^
*χ*^2^/*t*					6.0211
*P*					0.0141

^*∗*^Comparison with the CG group (*p* < 0.05).

## Data Availability

The data used to support the findings of this study are included within the article.
